# Gender Convergence in Alcohol Consumption Patterns: Findings from the Korea National Health and Nutrition Examination Survey 2007–2016

**DOI:** 10.3390/ijerph17249317

**Published:** 2020-12-13

**Authors:** Minkyung Kang, Ari Min, Haeyoung Min

**Affiliations:** 1 College of Nursing, Keimyung University, 1095 Dalgubeol-daero, Daegu 42601, Korea; minkyungkang@kmu.ac.kr; 2 Department of Nursing, Chung-Ang University, 84 Heukseok-ro, Seoul 06974, Korea; amin@cau.ac.kr; 3 College of Nursing, Gyeongsang National University, 816-15 Jinju-daero, Jinju 52727, Korea

**Keywords:** alcohol use, binge drinking, gender difference, Korean

## Abstract

Gender differences in alcohol use have narrowed over the decades. This study aimed to explore changes in alcohol consumption patterns between 2007 and 2016 to identify gender convergence in alcohol use in Korea. Data from the Korea National Health and Nutrition Examination Survey were used. For all respondents (41,662 girls/women and 32,041 boys/men) aged ≥12 years, lifetime drinking, current drinking, age of drinking onset, heavy alcohol use, and binge drinking were analyzed. Gender differences in heavy alcohol use and binge drinking decreased from 2007 to 2016 (*p* = 0.001 and *p* < 0.001, respectively). The prevalence of heavy alcohol use and binge drinking decreased in boys/men (67.5% to 63.9%, *p* = 0.001; 63.4% to 60.9%, *p* = 0.001, respectively), but not in girls/women (50.2% to 50.4%, *p* = 0.279; 30.6% to 32.0%, *p* = 0.994, respectively). The proportion of lifetime abstainers decreased among both girls/women (24.3% to 19.1%, *p* < 0.001) and boys/men (12.1% to 9.7%, *p* = 0.01). In girls/women, the mean age of drinking onset decreased (from 24.1 to 23.6 years, *p* = 0.017); however, in boys/men, significant changes were not observed (from 18.9 to 18.7 years, *p* = 0.307). Healthcare providers should be aware of the growing health risks resulting from increased unhealthy alcohol use in women and develop gender-specific preventive interventions.

## 1. Introduction

Worldwide, men consume more alcohol than women [1]. However, gender differences in this pattern of alcohol use, including problem drinking, have narrowed over recent decades [2,3,4,5,6]. According to studies conducted in the United States, the magnitude of the increase in alcohol use frequency in women was twice that in men between 2001–2002 and 2012–2013 [5], and the prevalence of binge drinking significantly increased in women, but not in men, between 2000 and 2016 [6]. Similarly, the prevalence of binge drinking increased among Korean women (20.5% to 26.9%), but decreased among men (53.7.1% to 50.8%) between 2007 and 2018 [7,8]. Moreover, on average, Korean men and women consume about six drinks per occasion [9], which is much greater than the low-risk drinking limits (<3 drinks per day for men, <2 drinks per day for women) recommended by the Korea Disease Control and Prevention Agency. [10].

While men are known to experience more alcohol-related problems relative to women [11], the latter are more vulnerable to the adverse effects of alcohol, such as cancer risk or cardiovascular disease [12,13]. Because women have a lower volume of body water and more body fat relative to men, they experience higher blood alcohol levels when age-matched men and women consume equal amounts of alcohol [14]. Studies have reported the negative effects of alcohol consumption on fertility in both men [15] and women [16]. Moreover, alcohol consumption in women of reproductive age and pregnant women has the potential to harm maternal and fetal health through issues such as impaired fertility and fetal alcohol spectrum disorder [17]. Understanding gender differences in alcohol consumption in Korea would be important to develop gender-specific preventive interventions or to change the current public initiatives to reduce alcohol-related health problems.

Despite the increase in alcohol use and vulnerability to the adverse effects of alcohol in women, limited studies have explored gender-specific changes in alcohol use patterns. Gender differences in patterns of alcohol use exist generally, but the sizes of these differences vary according to population and culture [1]. For example, men’s drinking rates were higher than those of women in Australia, the United States, and Korea, but the gender difference was largest in Korea [18]. To our knowledge, few studies have examined trends in changes in alcohol use patterns for each gender over time in the Korean population. Previous studies conducted in Korea examined gender-specific alcohol drinking patterns using a cross-sectional study design [19], or explored time trends in alcohol use comparing two specific years [20]. Only one study examined the seven-year trend of alcohol use, although it was limited to harmful alcohol use and adults aged 20–64 years [3]. Therefore, the present study aimed to explore changes in patterns of alcohol use, including lifetime abstinence, current drinking, age of drinking onset, heavy alcohol use, and binge drinking, in Korean girls/women and boys/men between 2007 and 2016 to identify gender convergence in alcohol use. Additionally, we explored the contributing factors of gender convergence in patterns of alcohol use.

## 2. Materials and Methods

### 2.1. Data Source and Study Sample

Weighted data from the 2007 to 2016 Korea National Health and Nutrition Examination Survey (KNHANES) were used to assess prevalence rates, means, and trends in alcohol use for girls/women and boys/men. The KNHANES is a cross-sectional nationwide survey of non-institutionalized civilian populations aged ≥1 year and conducted annually by the Korea Centers for Disease Control and Prevention. The survey consists of health interviews, health examinations, and nutrition surveys, which are administered in face-to-face interviews on site. From 2008 to 2016, about 10,000 individuals were surveyed each year (ranging from 12,528 in 2008 to 10,806 in 2016), except for in 2007, when 6455 individuals participated because the survey was conducted from July to December. In total, 74,063 participants (41,662 girls/women and 32,401 boys/men) aged ≥12 years were included in the current analysis. Although the minimum drinking age in Korea is 19 years, adolescents aged 12–18 years were included to explore trends in patterns of alcohol use in all age groups. The response rate was approximately 78.8% between 2007 and 2016.

### 2.2. Study Variables

Self-reported questionnaires were used to assess the patterns of alcohol use for each participant aged ≥12 years, except for binge drinking. The KNHANES collects data on binge drinking from only participants aged ≥19 years. Lifetime abstainers were defined as those who never consumed alcohol in their lifetime. The World Health Organization [21] defines current drinkers as those who have consumed at least one alcoholic drink in the last 12 months. The age at drinking onset in drinkers was defined as the age at which they first drank. The National Institute on Alcohol Abuse and Alcoholism [22] defines heavy alcohol use as consuming three or more drinks for girls/women and five or more drinks for boys/men on any day. Binge drinking was defined as consuming five or more drinks for girls/women and seven or more drinks for boys/men on the same occasion, with at least one or more binge drinking episodes per month. A standard drink is defined as roughly 10 g of pure alcohol, which is equivalent to 50 mL of soju (distilled spirit, 18–24% alcohol) and 250 mL of regular beer (4–5% alcohol) [10]. Potential confounding variables, such as age, household income, education, employment status, marital status, and pregnancy were taken into account.

### 2.3. Statistical Analysis

STATA version 13 (STATA Corp LP, College Station, TX, USA) was used to analyze the complex sample design of the KNHANES. Missing data were excluded from the data analyses once the prevalence rate and mean were calculated. The percentages of missing values of the study variables are presented in Supplementary Table S1. The prevalence rates, mean scores, and trends among girls/women and boys/men were calculated for lifetime abstinence, current drinking, age at drinking onset, heavy alcohol use, and binge drinking for each age group. Temporal trends in alcohol use according to age group (≥12 years (total), 12–18, 19–24, 25–34, 35–44, 45–54, 55–64, and ≥65 years) were assessed for girls/women and boys/men. In this stage, we did not control for participants’ demographic characteristics, because we mainly focused on the changes in patterns of alcohol use over time in girls/women and boys/men according to age groups. Linear regression was performed for continuous dependent variables, and logistic regression was performed for binary dependent variables to test for linear trends and gender differences. An interaction term between gender and survey year was included to test for changing gender differences between survey years in the models. An additional logistic regression was performed for significant alcohol-related variables to examine the gender convergence after controlling for confounding variables. Lastly, we performed a multivariate logistic regression analysis to further evaluate the odds ratios (ORs) for alcohol-related variables that showed significance according to gender, with age, household income, education, employment status, marital status, and pregnancy as confounding variables.

## 3. Results

### 3.1. Changes in Gender Differences in Alcohol Consumption by Age Group

Table 1 shows the changes in gender differences in alcohol consumption by age group. Between 2007 and 2016, the proportion of participants aged ≥12 years who had never consumed alcohol significantly decreased for both girls/women (24.3% to 19.1%, *p* < 0.001) and boys/men (12.1% to 9.7%, *p* = 0.001). The prevalence of lifetime abstinence significantly decreased in both men and women aged 55–64 and ≥65 years and in women aged 35–44 and 45–54 years. Gender gaps in the prevalence of lifetime abstinence significantly increased in those aged 45–54 years (*p* = 0.024).

The proportion of participants aged ≥12 years who had participated in at least one drinking occasion in the last 12 months did not change for girls/women (49.3% to 51.6%, *p* = 0.211) or boys/men (74.8% to 76.1%, *p* = 0.430). The proportion of current drinkers remained the same for both genders in all age groups over time, with the exception of a decrease observed in men aged 45–54 years (80.1% to 78.6%, *p* = 0.043). In addition, gender differences in prevalence rates for current drinking significantly decreased in those aged 45–54 years (*p* = 0.020).

The mean age at drinking onset for participants aged ≥12 years who had consumed alcohol significantly decreased over time in girls/women (from 24.1 to 23.6 years, *p* = 0.017), but not boys/men (from 18.9 to 18.7 years, *p* = 0.307). A decrease in age at drinking onset over time was observed in the following age groups: 25–34, 35–44, 45–54, and 55–64 years for women, and 35–44, 45–54, 55–64, and ≥65 years for men; the magnitude of the decrease in girls/women was relatively greater than that observed in men. Gender gaps in mean ages at drinking onset significantly increased in the following age groups: 19–24 (*p* = 0.012), 25–34, 35–44, 45–54, and 55–64 years (*p <* 0.001).

**Figure 1:**
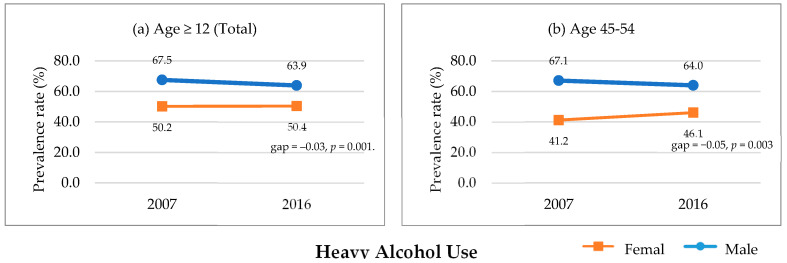
Significant gender convergence in heavy alcohol use, KNHANES 2007–2016. *Note.* KNHANES = Korea National Health and Nutrition Examination Survey. Annual prevalence rate of heavy alcohol use comparison between 2007–2016. Statistically significant changes in gender gap among (**a**) aged ≥12 (total) and (**b**) 45–55 were presented; in other age groups, significant changes were not found.

The proportion of participants aged ≥12 years who engaged in heavy alcohol use did not change significantly over time for girls/women (50.2% to 50.4%, *p =* 0.279), but significantly decreased for boys/men (67.5% to 63.9%, *p* < 0.001). The prevalence of heavy alcohol use significantly increased over time in women aged 55–64 years (*p* = 0.008), but significantly decreased in men aged 19–24 (*p* = 0.011), 25–34 (*p* = 0.023), and 45–54 years (*p* = 0.008). Gender gaps in the prevalence of heavy alcohol use significantly decreased in those aged ≥12 years (total; *p =* 0.001; Figure 1a) and 45–54 years (*p* = 0.003; Figure 1b).

**Figure 2:**
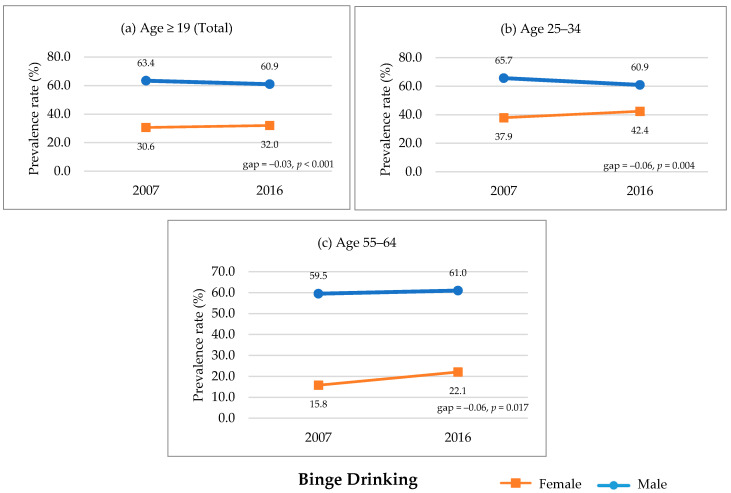
Significant gender convergence in binge drinking, KNHANES 2007–2016. *Note.* KNHANES = Korea National Health and Nutrition Examination Survey. Annual prevalence rate of binge drinking comparison between 2007 and 2016. Statistically significant changes in gender gap among (**a**) aged ≥19 (total), (**b**) 25–34, and (**c**) 55–64 were presented; in other age groups, significant changes were not found.

The proportion of participants aged ≥19 years who engaged in binge drinking did not change significantly for women (30.6% to 32.0%, *p* = 0.994), but it significantly decreased for men (63.4% to 60.9%, *p* < 0.001). The prevalence of binge drinking significantly decreased in men aged 25–34 years and 45–54 years. Gender differences in prevalence rates for binge drinking were significantly reduced in those aged ≥19 (total; *p <* 0.001; Figure 2a), 25–34 years (*p =* 0.004; Figure 2b), and 55–64 years (*p =* 0.017; Figure 2c).

### 3.2. Factors Associated with Heavy Alcohol Use and Binge Drinking

After controlling for possible confounding variables (i.e., household income, education, employment status, marital status, and pregnancy), no significant gender differences were observed for heavy alcohol use and binge drinking. That is, the cofounding variables may have led to significant gender convergence in alcohol-related variables (detailed results are not presented in this paper).

The results of multivariate logistic regression models that evaluated different effects of confounding variables on alcohol use by gender are presented in Table 2. The highest ORs of heavy alcohol use and binge drinking were observed in both women and men aged 19–24 years and 35–34 years. Household income ≤24th percentile was a risk factor for heavy alcohol use in women (OR = 1.37, 95% CI: 1.78–2.06). Household income ≤24th percentile (OR = 1.32, 95% CI: 4.13–1.56) and 25–49th percentile (OR = 1.14, 95% CI: 1.01–1.29) were risk factors for binge drinking in women. However, household income was not a risk factor of heavy alcohol use and binge drinking in men. Among women, those with a low education level had an approximately two-fold higher risk of heavy alcohol use and binge drinking compared to college graduates. Among men, high school graduates had an increased risk of heavy alcohol use (OR = 1.13, 95% CI: 1.02–1.25), and men with a low education level had a higher risk of binge drinking than college graduates, similar to women. Regarding employment status, the ORs of heavy alcohol use were similar to those of binge drinking, regardless of gender. Employed women (OR = 1.26, CI: 1.16–1.36) and men (OR = 1.21, CI: 1.09–1.35) had increased risks of both heavy alcohol use and binge drinking compared to unemployed individuals. Regarding marital status, married women had a decreased risk of both heavy alcohol use (OR = 0.57, 95% CI: 0.48–0.66) and binge drinking (OR = 0.65, 95% CI: 0.55–0.76). In contrast, married men had an increased risk of binge drinking (OR = 1.18, 95% CI: 1.03–1.35). Pregnancy was not associated with either heavy alcohol use or binge drinking in women.

## 4. Discussion

The main finding of the study was that gender differences in patterns of alcohol use, particularly with respect to the prevalence of heavy alcohol use and binge drinking, decreased from 2007 to 2016 in the Korean population. Korean girls/women began consuming alcohol at a younger age, and the prevalence of heavy alcohol use and binge drinking was not reduced. In contrast, Korean boys/men altered their alcohol use patterns in positive ways, reducing the prevalence of heavy alcohol use and binge drinking, although their age of drinking onset did not change. The proportion of lifetime abstainers showed a greater decline among girls/women relative to that among boys/men, and these rates remained relatively stable in current drinkers. These changes led to a convergence of patterns of alcohol use in Korean boys/men and girls/women during the last decade.

To our knowledge, few studies have examined changes in alcohol use among men and women over time. Previous studies from multiple countries reported that boys/men consume more alcohol than girls/women, drink more frequently, are more likely to be hazardous drinkers, and experience more social problems resulting from drinking [23,24]. Notably, the current results showed that the size of gender gaps in several variables used to measure alcohol use patterns decreased in Korean boys/men and girls/women from 2007 to 2016. Similar trends in alcohol use patterns were observed in previous studies conducted in Norway [2], the United States [4], and Korea [3]. Between 1984 and 2008, Bratberg et al. [2] reported that, in Norwegians aged ≥20 years, rates of abstaining decreased more, and the proportions of recent drinkers and mean annual alcohol consumption increased more in women than in men. Furthermore, White et al. reported that the proportion of current drinkers (44.9% to 48.3%) and the number of drinking days per month increased (from 6.8 to 7.3 days) in women, but did not change (57.4% to 56.1%) or decreased (from 9.9 to 9.5 days) in men between 2002 and 2012 [4]. The prevalence of binge drinking increased (15.1% to 16.2%) in women, but not in men (31.1% to 30.1%) [4]. In a more recent study of men and pregnant and non-pregnant women in the United States, the amount of drinking and binge drinking decreased in men and pregnant women, while it increased in non-pregnant women aged 21–44 years [25]. In Koreans aged 20–64 years, the prevalence of harmful drinking (i.e., consuming > 60 g and > 40 g of alcohol per drinking day for men and women, respectively) decreased significantly in men (23.3% to 22.9%), but not in women (3.7% to 6.8%) between 2007 and 2014 [3]. Our findings are consistent with those of previous studies, suggesting that global gender differences in this pattern of alcohol use have decreased over time.

The reasons for the decrease in gender differences in alcohol use are unclear and complicated; however, they could involve recent trends in employment status, marital status, and pregnancy in women [4]. In this study, employed women had a higher risk of risky drinking behaviors, and married women had a lower risk compared to others. Furthermore, low levels of income and education were risk factors for alcohol consumption in women. Unexpectedly, pregnancy did not affect women’s drinking behavior in our study. White et al. reported that the prevalence rates for current and binge drinking were higher in women who had never married or were not pregnant and were employed, relative to their counterparts [4]. French et al. compared the socioeconomic correlates of risky drinking in middle-aged and older populations across various countries, including the United States and Korea [18]. They reported that risky drinking was associated with higher education levels in American women, while it was associated with being single in Korean women [18]. However, Choe et al. [3] found that being married and having a low income were risk factors for harmful drinking in Korean women. Since these study results are inconsistent, future studies should include various psychosocial and environmental factors that contribute to these changes to further clarify the possible reasons for the decrease in gender differences in alcohol use.

The number of employed women is increasing, and they often attend company dinners that involve drinking [26]. In addition, these women tend to experience work-related stress, which could lead to increased alcohol use as a form of relief [27]. On the contrary, there are either decreases or little change in alcohol use in men, which may imply decreased interest in drinking, shifts to other substances [25], or effects of interventions emphasizing men’s problem drinking. However, men still consume a higher level of alcohol than women, and little information exists regarding the factors affecting positive changes in their alcohol use.

Pregnancy status reduces women’s alcohol consumption, but approximately 9.8% (95% CI: 8.9% to 11.1%) of women reported consuming alcohol during pregnancy [28]. Furthermore, 2.3% of pregnant women reported binge drinking, and 0.4% reported heavy drinking despite the risk of harm to the fetus [29]. In 2016, the Korean government began to add a warning on alcohol bottles about the risk of birth defects for pregnant women to educate the public, specifically girls/women, about the dangers of alcohol consumption [30]. However, the rate of adding warning signs to alcohol bottles is too small to raise awareness among the public, and the effectiveness of the policy is doubtful so far.

Understanding gender-specific trends in alcohol use is crucial, because substantial changes in the gender distribution of alcohol use may mean that the current health policies, resource allocation, and strategies to reduce alcohol use and related problems may need to be reconsidered [31]. In fact, alcohol use and its disorders have generally been viewed as a male phenomenon [9,21], and gender has not been well-addressed in alcohol-related policy [32]. A systematic review of the effectiveness of alcohol policy interventions suggests that researchers and policymakers require further consideration of gender differences in the impact of population-level alcohol policy interventions, such as advertising controls and mass media campaigns [32]. Furthermore, the present study’s results suggest that healthcare providers should develop and implement gender-specific preventive education and interventions for alcohol use. Women of reproductive age need to be screened for and advised about harmful drinking and its increased risk for fetal alcohol spectrum disorder. Healthcare providers need to educate women about low-risk drinking and other health risks (e.g., breast cancer) from harmful drinking [33]. Recently, the U.S. Preventive Service Task Force recommended that heavy alcohol use be screened in primary care settings and that heavy drinkers be provided with brief behavioral counseling interventions [34]. However, as employed women tend to have limited access to healthcare services due to lack of time and the stigma attached to women’s alcohol abuse, employers may need to consider providing supportive interventions and education in the workplace [35]. Further research using well-controlled randomized clinical trials that examine the effects of gender-specific treatment is needed to develop effective strategies [24].

Policies for alcohol should be implemented that target the risky drinking patterns of a population. Currently, Korea has no existing policies regarding the purchasing time; stores in Korea are allowed to sell alcohol 24 h a day. Thus, people may drink at any time during the day. A study reported that females were about 2.2 times more likely to engage in problematic alcohol use after midnight compared to males [26]. Other countries, including the European countries, the United States, and Canada, have regulations that restrict stores to selling alcohol within permitted hours [36,37]. Considering the increases in heavy alcohol use and binge drinking in girls/women in Korea, a well-articulated policy that limits the permitted hours for selling alcohol to mitigate risky alcohol use is recommended.

This study has some limitations. The data for participants’ alcohol consumption were based on a self-report survey, which could therefore have led to recall bias and inaccuracy. For example, women tend to underestimate the content of alcohol units to a greater extent than men [25]. Therefore, more objective and accurate measures, such as blood samples, are required to enhance data accuracy for alcohol consumption. In addition, although the KNHANES includes representative national data for Korea with an acceptable response rate, these data cannot be used to determine causal relationships between alcohol consumption and related factors, such as individual, environmental, and social issues.

## 5. Conclusions

Patterns of alcohol use between 2007 and 2016 have changed according to gender, and long-lasting gender gaps in alcohol use decreased in the Korean population. Women consume more alcohol compared to women in previous years, and could experience greater alcohol-related problems than they did in the past, indicating that women’s alcohol use may be a growing health problem in Korea. Healthcare providers should disseminate information regarding emerging health risks resulting from increased unhealthy alcohol use in women and be aware of the importance of developing gender-specific preventive education and interventions for alcohol use.

## Figures and Tables

**Table 1:** Changes in gender differences in alcohol consumption by age group, KNHANES 2007–2016.

	Age Group	Female		Male		Gender Gap	
		Coefficient ^a^	*p*-Value	Coefficient ^a^	*p*-Value	Coefficient ^b^	*p*-Value
**Lifetime abstinence**	≥12 (total)	−0.03	<0.001	−0.02	0.001	0.01	0.568
	12–18	0.04	0.066	0.03	0.058	−0.01	0.758
	19–24	0.03	0.564	−0.04	0.530	−0.06	0.388
	25–34	−0.04	0.253	0.01	0.876	0.04	0.405
	35–44	−0.09	<0.001	−0.03	0.376	0.05	0.249
	45–54	−0.08	<0.001	−0.001	0.974	0.08	0.024
	55–64	−0.09	<0.001	−0.07	0.011	0.01	0.664
	≥65	−0.03	0.005	−0.04	0.031	−0.01	0.557
**Current drinking**	≥12 (total)	0.01	0.211	−0.01	0.430	−0.01	0.135
	12–18	0.01	0.778	0.03	0.151	0.02	0.550
	19–24	0.002	0.908	−0.01	0.631	−0.02	0.653
	25–34	0.01	0.275	−0.01	0.577	−0.03	0.304
	35–44	0.01	0.213	−0.01	0.479	−0.03	0.197
	45–54	0.01	0.291	−0.03	0.043	−0.04	0.020
	55–64	0.02	0.234	0.001	0.924	−0.02	0.467
	≥65	0.01	0.279	0.01	0.363	−0.001	0.927
**Age at drinking onset**	≥12 (total)	−0.07	0.017	−0.01	0.307	0.06	0.051
	12–18	0.08	0.009	0.05	0.049	−0.03	0.469
	19–24	0.04	0.070	0.11	<0.001	0.08	0.012
	25–34	−0.07	<0.001	0.04	0.050	0.11	<0.001
	35–44	−0.25	<0.001	−0.09	<0.001	0.16	<0.001
	45–54	−0.55	<0.001	−0.11	<0.001	0.44	<0.001
	55–64	−0.56	<0.001	−0.10	0.019	0.45	<0.001
	≥65	−0.003	0.969	−0.13	0.009	−0.12	0.261
**Heavy alcohol use**	≥12 (total)	−0.01	0.279	−0.03	<0.001	−0.03	0.001
	12–18	−0.02	0.613	−0.05	0.087	−0.03	0.502
	19–24	−0.01	0.566	−0.06	0.011	−0.05	0.140
	25–34	−0.004	0.734	−0.04	0.023	−0.03	0.106
	35–44	−0.001	0.953	−0.03	0.061	−0.02	0.196
	45–54	0.02	0.181	−0.04	0.008	−0.05	0.003
	55–64	0.04	0.008	0.03	0.059	−0.02	0.368
	≥65	−0.01	0.742	−0.02	0.271	−0.01	0.729
**Binge drinking**	≥19 (total)	−0.0001	0.994	−0.03	<0.001	−0.03	<0.001
	19–24	−0.01	0.701	−0.04	0.079	−0.03	0.301
	25–34	0.02	0.095	−0.04	0.023	−0.06	0.004
	35–44	0.003	0.799	−0.02	0.184	−0.02	0.263
	45–54	0.0003	0.981	−0.04	0.015	−0.03	0.056
	55–64	0.04	0.068	−0.02	0.148	−0.06	0.017
	≥65	0.02	0.527	−0.01	0.354	−0.03	0.333

*Note.* KNHANES = Korea National Health and Nutrition Examination Survey. ^a^ Trend of Changes in Annual Mean or Prevalence Rate of Alcohol Consumption from 2007 to 2016; ^b^ Trend of Changes in Gender Gap from 2007 to 2016.

**Table 2:** Odds ratios for heavy alcohol use and binge drinking according to gender, KNHANES 2007–2016.

Variable	Category	Heavy Alcohol Use		Binge Drinking	
		Female	Male	Female	Male
		OR (95% CI)	OR (95% CI)	OR (95% CI)	OR (95% CI)
**Age range (years)**	12–18	4.39 (3.17–6.08)	3.60 (2.73–4.47)	2.28 (1.42–3.65)	0.85 (0.58–1.23)
	19–24	11.29 (8.66–14.72)	7.15 (5.55–9.23)	8.60 (6.44–11.49)	3.35 (2.65–4.22)
	25–34	8.00 (6.45–9.92)	6.24 (5.18–7.52)	9.66 (7.47–12.48)	3.12 (2.61–3.73)
	35–44	5.65 (4.62–6.91)	5.02 (4.30–5.86)	6.40 (5.00–8.19)	3.01 (2.59–3.49)
	45–54	4.20 (3.48–5.07)	4.30 (3.71–4.98)	4.86 (3.85–6.15)	2.85 (2.46–3.29)
	55–64	2.19 (1.82–2.63)	2.60 (2.28–2.97)	2.31 (1.83–2.92)	2.15 (1.87–2.46)
	≥65	reference	reference	reference	reference
**Household income**	≤24th percentile	1.37 (1.78–2.06)	1.04 (0.90–1.20)	1.32 (4.13–1.56)	1.10 (0.95–1.27)
	25–49th percentile	1.11 (0.99–1.24)	0.98 (0.88–1.10)	1.14 (1.01–1.29)	0.91 (0.81–1.02)
	50–74th percentile	1.11 (1.00–1.23)	0.95 (0.85–1.05)	1.07 (0.96–1.20)	0.92 (0.82–1.02)
	≥75th percentile	reference	reference	reference	reference
**Education**	Elementary school	1.74 (1.48–2.06)	1.00 (0.86–1.17)	2.33 (1.92–2.84)	1.33 (1.15–1.55)
	Middle school	1.72 (1.47–2.01)	0.98 (0.85–1.14)	2.65 (2.22–3.17)	1.26 (1.08–1.46)
	High school	1.79 (1.62–1.98)	1.13 (1.02–1.25)	2.03 (1.82–2.26)	1.17 (1.06–1.30)
	≥College	reference	reference	reference	reference
**Employment status**	Yes	1.26 (1.16–1.36)	1.21 (1.09–1.35)	1.30 (1.19–1.42)	1.38 (1.24–1.54)
	No	reference	reference	reference	reference
**Marital status**	Yes	0.57 (0.48–0.66)	1.11 (0.95–1.30)	0.65 (0.55–0.76)	1.18 (1.03–1.35)
	No	reference	reference	reference	reference
**Pregnancy**	Yes	0.96 (0.63–1.47)		1.05 (0.66–1.67)	
	No	reference		reference	

*Note.* CI = Confidence interval, KNHANES = Korea National Health and Nutrition Examination Survey.

## Supplementary Materials

The following are available online at https://www.mdpi.com/1660-4601/17/24/9317/s1, Table S1: Percentage of missing values for study variables, KNHANES 2007–2016.
