# Supplementary material for: Gender Convergence in Alcohol Consumption Patterns: Findings from the Korea National Health and Nutrition Examination Survey 2007–2016
Source: Int J Environ Res Public Health. 2020 Dec 13;17(24):9317. doi: 10.3390/ijerph17249317 (PMC7764704; doi:10.3390/ijerph17249317)
Supplement: Supplementary file 1 [file ijerph-17-09317-s001.pdf]

## Supplementary Materials

**Table S1.** Percentage of missing values for study variables, KNHANES 2007–2016.

| Variable | Total (%) | Missing value (%) | | | | | | | | | |
|---|---|---|---|---|---|---|---|---|---|---|---|
| | | **2007** | **2008** | **2009** | **2010** | **2011** | **2012** | **2013** | **2014** | **2015** | **2016** |
| **Lifetime abstinence** | 3.21 | 0.14 | 0.20 | 0.36 | 1.39 | 3.06 | 6.35 | 7.71 | 8.19 | 3.64 | 1.87 |
| Current drinking | 19.05 | 18.37 | 16.83 | 17.90 | 18.35 | 19.30 | 21.66 | 22.56 | 22.12 | 18.00 | 16.02 |
| Age at drinking onset | 19.44 | 22.94 | 18.63 | 17.93 | 17.60 | 19.31 | 21.83 | 22.73 | 22.32 | 18.16 | 16.20 |
| Heavy alcohol use | 31.36 | 31.78 | 30.00 | 30.32 | 28.33 | 30.35 | 33.88 | 35.38 | 34.54 | 30.91 | 29.18 |
| Binge drinking | 33.31 | 34.84 | 33.88 | 33.33 | 31.64 | 33.32 | 36.97 | 35.42 | 34.54 | 30.91 | 29.18 |
